# Profile of medical students in the first group of the *Faculdade Israelita de Ciências da Saúde Albert Einstein*

**DOI:** 10.1590/S1679-45082018AO4228

**Published:** 2018-09-11

**Authors:** Ângela Tavares Paes, Bruna de Freitas Dias, Giulia Nicolucci Eleutério, Vitória Penido de Paula

**Affiliations:** 1 Faculdade Israelita de Ciências da Saúde Albert Einstein, Hospital Israelita Albert Einstein, São Paulo, SP, Brazil

**Keywords:** Students, Medical, Education, Medical, undergraduate, Estudantes de Medicina, Educação de graduação em Medicina

## Abstract

**Objective:**

To describe first medical students' profile of the *Faculdade Israelita de Ciências da Saúde Albert Einstein.*

**Methods:**

Data were collected using an electronic questionnaire during the Biostatistics course in August of 2016. The students were inquired about demographic characteristics, data on their secondary education and college entrance exams, practice of physical exercise, leisure activities done, to have a physician in the family, and specialty that they intended to pursue as a career.

**Results:**

Most of the students were women aged 18 to 21 years and who were originally from the state of São Paulo, had received secondary education in a private school, took a course to prepare for college entrance exam, and participated in more than 5 college entrance exams in the same year they entered in the School of Medicine. The majority of participants practiced physical exercise regularly and were engaged in common leisure activities. Most of students (58%) had a physician in the family and more than half (52%) did not know which specialty to pursue as career. There was no association between relationship with a physician and the student's choice of a specialty (p=0.390).

**Conclusion:**

Although it was the first group of student of School of Medicine at *Faculdade Israelita de Ciências da Saúde Albert Einstein* who took a different admission process, our data showed that students' profile is similar to students from other colleges.

## RESUMO

**Objetivo:**

Descrever o perfil dos estudantes de Medicina ingressantes na primeira turma do curso de graduação em Medicina da Faculdade Israelita de Ciências da Saúde Albert Einstein.

**Métodos:**

Os dados foram coletados a partir de uma pesquisa realizada pela internet durante o curso de Bioestatística, em agosto de 2016. A pesquisa abordou características sociodemográficas, dados do ensino médio e dos vestibulares, prática de atividade física, lazer, parentesco médico e especialidade que pretendia seguir.

**Resultados:**

A maioria dos estudantes de Medicina era do sexo feminino, procedente do Estado de São Paulo, com idade entre 18 a 21 anos, estudou o Ensino Médio em escola particular, fez cursinho pré-vestibular e prestou mais de cinco vestibulares no ano de ingresso na faculdade. Em geral, realizavam atividade física regularmente e tinham atividades de lazer comuns à essa faixa etária. A maior parte (58%) tinha algum parentesco médico e mais da metade (52%) não sabia qual especialidade seguir. Não houve associação entre parentesco médico e escolha da especialidade (p = 0,390).

**Conclusão:**

Apesar de ser a primeira turma e de ter passado por um processo seletivo diferenciado, os dados mostraram que o perfil dos alunos era semelhante ao encontrado em outras faculdades.

## INTRODUCTION

In Brazil in last year of secondary school many students are preparing to the vestibular - an entrance exam used by the Brazilian universities for their admission processes. In addition, at this time, students have to decide what course and college or university to attend. Among other options, the medical course in Brazil has one of the highest demand and, therefore, it is highly competitive for applicants to get into.

In general, a student entering in a medical undergraduate course has high technical ability, but not always he/she has the adequate profile to pursue a medical career. According to the guidelines of medical course curriculum established by the Brazilian Ministry of Education, a student should leave school of medicine with a general knowledge including issues related with humanization, critical, reflexive and ethical thinking, he/she must have the ability to work in different levels of health care promotion, prevention and recovery actions. In addition, students must understand health rehabilitation in an individual and collective perspective, his/her social responsibility and commitment with citizenship defense of human dignity, and delivery integral human-centered health care and promote a clinical practice always focused on social determinants of health and disease.^(^ ^1^ ^)^ This guideline suggests that medical students must be exposed to more than comprehensive and technical knowledge that is part of medical course curriculum.

After investigate admission processes of the main medical courses in Brazil, we observed that a variety of entrance exam types exist with different tests occurring in a single phase test, such as in the Marilia Medical School (FAMEMA) and Faculdade de Medicina de Ribeirão Preto (FAMERP), both which accept the Brazil's Exame Nacional do Ensino Médio (ENEM) as entrance exam. And those with double phase test such as Universidade Estadual de Campinas (UNICAMP), Universidade de São Paulo (USP) Universidade Estadual Paulista “Júlio de Mesquita Filho” (UNESP), Universidade Federal de São Paulo (UNIFESP) and the Faculdade Israelita de Ciências da Saúde Albert Einstein (FICSAE). Exams can include multiple choices questions in the first phase and open-ended questions in the second phase. In addition, some entrance exam, especially those adopting a single day test, can include more than one type of question, and these exams can also include argumentative essay or different type of texts writing. Although many efforts are made to select students with higher critical ability, medical school entrance exams in Brazil often use selection methods that prioritize measurement of applicants’ cognitive skills.

In 2016, the FICSAE started a medical undergraduate course with a proposal of new teaching method and a different admission process that uses a non-cognitive assessment to assess applicants’ skills such as critical thinking, teamwork, communication, motivation and ethics. This admission process includes two phases: the first phase involves the assessment of academic competence using objective tests and open-ended questions,^(^ ^2^ ^)^ and the second phase the approved applicants in the first phase undergo multiple mini interviews (MMI).^(^ ^3^ ^)^ Multiple mini interviews are structured time-controlled interviews. This type of admission, which is a new approach in Brazil, is currently used in Canada and United Kingdom, and in more than 30 medical schools in the United States. The aim of the method is to highlight non-cognitive abilities in both human and professional aspect by provide more value to competency and passion, effective communication, empathy, critical thinking, ethics, leadership, teamwork and motivation.^(^ ^4^ ^)^

In addition to differentiated admissions criteria, the FICSAE uses as main the teaching method the team-based learning (TBL).^(^ ^5^ ^,^ ^6^ ^)^ The TBL involves students proactivity in which they need to come to class with previous studied content, and team activities with emphasis on the medical practice. Although these active method would be present in most of the medical school, the TBL specifically, still little known. Of 52 medical schools in São Paulo State, 19 (36%) describes in pedagogy project the use of the active methodologies. Of these, 10 (52.6%) adopted the problem-based learning (PBL),^(^ ^7^ ^)^ which is difference of TBL in a number of aspects, such as existence of readiness assurance test and supervision done by a single faculty member to all student groups.

The characteristics of the admission process and teaching method aligned with fact that starting of a new undergraduate course turn interesting to determine the profile of entrance students. The description of students’ profile can provide interesting information for both general and education professionals audience.

## OBJECTIVE

To describe profile of the first year students of the School of Medicine at *Faculdade Israelita de Ciências da Saúde Albert Einstein* by considering sociodemographic characteristics, information from students’ secondary education, entrance exams, practice of physical exercise, leisure activities done, having a physician in the family, specialty that they intend to pursue and availability to participate in academic activities.

## METHODS

This was a cross-sectional descriptive study with a quantitative approach. We included 50 students who made up the first student group admitted in the School of Medicine at FICSAE, which started in the first semester of 2016.

A Google form was created to collect opinion. The form was fulfilled by students who volunteered during a biostatistics class. The first proposed questionnaire included multiple open-ended questions, but it was difficult for data processing. After organization a database by creating a forum for discussion, students provided feedback and proposed improvements. The second questionnaire included 23 questions (Appendix 1). This second questionnaire was applied again, and data collected were used during class as an example to introduce the concept of descriptive statistics. All respondents of the study signed a consent form. Ethics committee approval was not required for our study based on resolution 510/16.

## RESULTS

**Figure 1:**
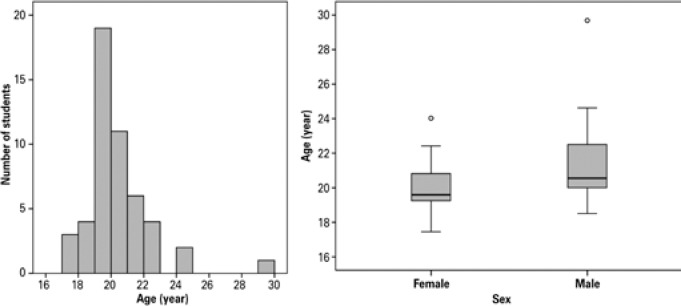
Distribution of new students by age (in years) and sex

Sociodemographic characteristics and information of candidates before admission in the school of medicine are described in table 1 and 2. The majority of applicants included were women (74%). Students’ mean age was 20.3 years (standard deviation - SD=2.1) and they age ranged between 17 and 29 years (Figure 1). The majority of students (80%) was from Southeast region, 76% was from São Paulo State, and 23 students (46%) were born in the city of São Paulo. Of those who was born in other cities, 10 moved from their home city to study for the entrance exam. Most of students were catholic (42%) and 11 (22%) did not have religion.

**Table 1:** Sociodemographic characteristics of candidates

Variable		n (%)
Sex		
	Man	13 (26.0)
	Woman	37 (74.0)
Age range		
	Younger than 18 years	3 (6.0)
	Between 18 and 21 years	34 (68.0)
	Older than 21 years	13 (26.0)
State of origin		
	São Paulo	38 (76.0)
	Other states	12 (24.0)
	Region of origin	
	Southeast*	40 (80.0)
	Central-West†	7 (14.0)
	South‡	2 (4.0)
	Northeast§	1 (2.0)
Religion		
	Catholic	21 (42.0)
	No religion	11 (22.0)
	Atheism	3(6.0)
	Spiritist	2 (4.0)
	Protestant	1 (2.0)
	Paganism	1 (2.0)
	Candomblé - Afro-American religion	1 (2.0)
	Evangelist	1 (2.0)
	No information	9 (18.0)

* 38 were from São Paulo and 2 from Minas Gerais;

† 4 from Goias; 2 from Mato Grosso and 1 from Mato Grosso do Sul;

‡ 1 from Parana and 1 from Rio Grande do Sul;

§ Maranhão.

**Table 2:** Data of applicants on secondary education and entrance exam

Variables		n (%)
Type of school in which the student attended the secondary education		
	Private	49 (98.0)
	Part in public and part in private school	1 (2.0)
Time - in years - since the conclusion of the secondary education		
	1	8 (16.0)
	2	17 (34.0)
	3	10 (20.0)
	4	8 (16.0)
	5 or more	7 (14.0)
Does student attend preparatory course for entrance exam?		
	Yes	43 (86.0)
	No	7 (14.0)
Number of entrance exam done*		
	Less than 4	–
	4	2 (4.0)
	5 or more	48 (96.0)
What entrance exam you took in addition to the FICSAE† exam?		
	FUVEST	46 (92.0)
	UNICAMP	43 (86.0)
	UNIFESP	42 (84.0)
	UNESP	40 (80.0)
	Other São Paulo States with its own entrance exam	34 (68.0)
	Other public universities/colleges out of the São Paulo State	26 (52.0)
	Other universities/colleges in São Paulo State that use the *Sisu* score - a unified selection system in Brazil	14 (28.0)
	Other private universities/colleges out of the São Paulo State	7 (14.0)

* In the year of admission;

† multiple answer.

FICSAE: Faculdade Israelita de Ciências da Saúde Albert Einstein; FUVEST: Fundação Universitária para o Vestibular; UNICAMP: Universidade Estadual de Campinas; UNIFESP: Universidade Federal de São Paulo; UNESP: Universidade Estadual Paulista “Júlio de Mesquita Filho”.

Forty-nine students (98%) studied in private schools. On average, students had concluded secondary school 3 years ago (SD=1.9), only seven (14%) did not use pre admission exam and all took at least four other entrance exams. The mainly college entrance exams done by students were FUVEST (92%), UNICAMP (86%), and UNIFESP (84%).

**Figure 2:**
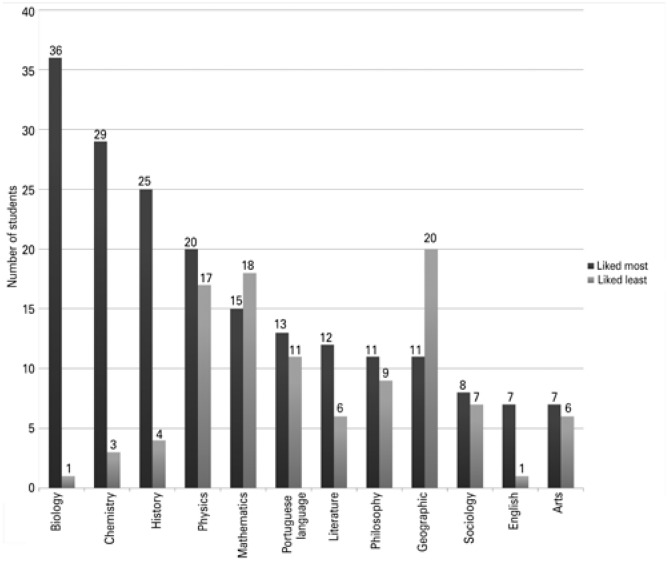
Disciplines that students liked most and least during secondary education

When students were inquired about subjects they liked most and least in secondary education (Figure 2), most liked subjects were Biology (72%) and Chemistry (58%). We also observed that most of students mentioned History as of the subject that they liked most. Among courses that students liked least were geographic (40%), mathematics (36%) and Physics (34%), however, the two latter were also mentioned as subjects that some of them liked most.

Among students, 64% reported to do some physical exercise, and 38% of them exercise two or three times a week (Table 3). Most of them exercise in a gym, 54% did weight training or collective exercise, and 24% did run-walk exercise. In terms of leisure activities, 41 students mentioned sleeping (82%), go to the movies (72%), and watching TV (68%).

**Table 3:** Data on physical exercise and leisure activities of new students

Variables		n (%)
Physical exercise		
	Yes	32 (64.0)
	No	18 (36.0)
Frequency of physical exercise per week		
	Up to once weekly	7 (14.0)
	2-3 times a week	19 (38.0)
	≥4 times a week	6 (12.0)
What physical exercise do you do?		
	Gym (weight training /collective exercise)	27 (54.0)
	Running/walking	12 (24.0)
	Swimming	3(6.0)
	Soccer	2 (4.0)
	Tennis	2 (4.0)
	Dance	1 (2.0)
	Handball	1 (2.0)
	Other	1 (2.0)
	Fighting (boxing and taekwondo)	1 (2.0)
	Pilates	1 (2.0)
Leisure activities		
	Sleeping	41 (82.0)
	Go to the movies	36 (72.0)
	Watching TV	34 (68.0)
	Dance club (bars/night clubs)	31 (62.0)
	Reading	23 (46.0)
	Parks	19 (38.0)
	Go shopping	19 (38.0)
	Sports	18 (36.0)
	Visit relatives	17 (34.0)
	Date with boyfriend/girlfriend	16 (32.0)
	Live shows	16 (32.0)

Almost 60% of students had one physician in the family, and for 24% of them the mother or father was physician. We observed that most students who had a physician in the family were those living in different region from the Southeast (8/10 = 80%).

More than half of students (52%) did not know what specialty they would pretend to pursue in medical career, among those who already know, the choice varied (Table 4). The choice of the specialty (to know or not) was not associated with the specialty of the physician in the family (χ^2^ test; p=0.390).

**Table 4:** Relationship of students with medicine

Variables		n (%)
To have a physician in the family		
	Yes	29 (58.0)
	No	21 (42.0)
Students' family ties with a physician		
	Father/mother	12 (24.0)
	Uncle/Aunt	10 (20.0)
	Other	9 (18.0)
	Grandparents	5 (10.0)
Medical specialty that students wished to pursue as a medical career		
	Do not know	26 (52.0)
	Oncology	4 (8.0)
	Psychiatry	4 (8.0)
	Cardiology	2 (4.0)
	Medical genetics	2 (4.0)
	Neurosurgery	2 (4.0)
	Pediatrics	2 (4.0)
	Surgery	1 (2.0)
	Cardiovascular surgery	1 (2.0)
	Head and Neck Surgery	1 (2.0)
	Prenatal/pediatrics surgery	1 (2.0)
	Gastroenterology	1 (2.0)
	Gynecology and Obstetrics	1 (2.0)
	Legal and Forensic medicine	1 (2.0)
	Radiology and imaging diagnosis	1 (2.0)

## DISCUSSION

The decision to attend the first group of private School of medicine is not simple, especially for the insecurity to invest in a new school that was not evaluated in terms of quality by the national higher education assessment system, which is the case of FICSAE. Our school of medicine there has an extra challenge because of the new educational method and admission process adopted that, in a certain way, is subjective.

No specific characteristics were observed in the first group of new students at FICSAE that differed extensively from medical students from other universities.^(^ ^8^ ^–^ ^15^ ^)^

The majority of students were women - a fact not observed in previous studies.^(^ ^8^ ^–^ ^11^ ^)^ But our results agree with the growing tendency in Brazil in the last years of women seeking a career in medicine,^(^ ^16^ ^)^ however, in the students at FICSAE, we observed that, so far, there are no standard in terms of distribution among sexes. An evidence of this diversity is what was observed in the subsequent groups of new students in which the second group had 70.5% of women and third group had 46.2%.

For college education, students often seek institutions not so far from their home city. In our study, therefore, most of students were from the Southeast region. Although it was not part of the questionnaire, we inquired students from other regions about reasons to move to the city of São Paulo to attend college. The reason mentioned by students who did so was that in their region medical school of the State of São Paulo are well known for providing education with better quality compared with other states. In addition, some students reported not only family encouragement but also by coordinators of the secondary school they attended. Most of student who moved to São Paulo and had a physician in the family, and who were already in the career, reported that his/her relative encouragement also contributed to their decision of moving to São Paulo to study. The ease access to the capital of São Paulo by plane was also mentioned as a reason to choice the city for college education - which is not so ease in university or colleges in countryside.

An important fact concerning religion was that many students (28%) reported to be atheist or have no religion. This percentage was higher than what is observed among population from São Paulo (which proportion of atheists and those with no religion is 9% - according to the last census reported by the Brazilian Institute of Geography and Statistics - IBGE). The most common religion reported was Catholicism, an expected finding among Brazilian population that majority is catholic; around 60% of the population. Therefore, although FICSAE in a Jewish institution, this fact seemed not to influence the choice of students based on their religion preference.

Other expected data was that most of student attended secondary education in a private school. This expectation is given to the fact that students from private schools have higher access index in best colleges, and very competitive courses such as medicine. The number of previous attempts in entrance exams before formal entering in the school of medicine that was observed in our study is proof of level of difficulty of entrance exams for medical undergraduate courses.

We observed that most of students who had a physician in their family, and, different from what we expected, having a physician as a relative, did not influence the student's preference to pursue a specific medical specialty as a career.

Proportion of new students who did not know what specialty to follow was similar to finding reported in another study including students from fifth year of school of medicine.^(^ ^11^ ^)^

The preference of students for resting during leisure times can be justified because they were experiencing an intense period of dedication for studies. However, the practice of regular exercise was frequent in among students and higher than the frequency reported in a similar study carried out by medical students from other state.^(^ ^12^ ^)^

We highlight as limitations of this study the lack of investigating socioeconomic factors and other characteristics that might enhance the description of students’ profile. However, because the questionnaire was designed to be used in a practical activity in the classroom, no specific planning was done to provide a complete description of students’ profile. The decision of not inquiring about students’ family monthly income, which is common data in other studies,^(^ ^11^ ^–^ ^13^ ^)^ was because this information might cause some type of embarrassment to them considering that data was used during an exercise in the classroom of a biostatistics course. Although students’ economic status was not included, we believe that their family monthly income is not above the average because the school of medicine at FICSAE offers financing support or student loan, and approval for these supports are based on socioeconomic criteria not only in academic achievements.

We did not explore students’ expectations about medical profession, quality of the course, the active teaching-learning methodologies^(^ ^17^ ^)^ or data related with non-cognitive aspects - a fact of extreme value in admission process at FICSAE. Other studies can approach these aspects in the future.

Although assessment of mental health among medical students is currently a well discussed issue in the literature,^(^ ^18^ ^,^ ^19^ ^)^ we did not included questions on this subject because the first aim of our study was to be designed for a practical activity in the classroom. If our first aim was a scientific approach, the planning of the study would also entail instruments to approach students’ mental health given the fact that this discussion is relevance and interesting for academia and individuals involved with college admission process.

## CONCLUSION

Although this was the first group of the student of the *Faculdade Israelita de Ciências da Saúde Albert Einstein* and they went through an admission process that focused on non-cognitive skills, our findings show that students’ profile, based on characteristics investigated, was similar to medical students from others colleges.

## Appendix 1 Questionnaire created through Google forms

**Profile of students entering in medical course - 2016 class - 1**

This questionnaire has the aim to determine students' profile entering in the medical course of the Faculdade Israelita de Ciências da Saúde Albert Einstein. There is no need to Identify yourself on the form unless you wish to do so. Data collected will be used for didactic purposes only during the Scientific Method and Biostatistics class.

***Mandatory**

1. Sex
  Check all that apply.
  □ Male
  □ Female
2. Date of birth (day/month/year)*
  ____________________________________
  Example: 15/12/2012
3. State of Birth*
  Check only one.
  ◯ AL
  ◯ AP
  ◯ AM
  ◯ BA
  ◯ CE
  ◯ DF
  ◯ ES
  ◯ GO
  ◯ MA
  ◯ MT
  ◯ MG
  ◯ PA
  ◯ PB
  ◯ PR
  ◯ PE
  ◯ PI
  ◯ RJ
  ◯ RN
  ◯ RS
  ◯ RO
  ◯ RR
  ◯ SC
  ◯ SP
  ◯ SE
  ◯ TO
4. City of birth (if needed use lowercase/uppercase letter or word stress)*
  __________________________________
5. Type of school in which you attended the secondary education*
  Check only one.
  ◯ Private school
  ◯ Public school
  ◯ Part in public, and part in private school
6. What disciplines you liked most in the secondary education?*
  Check all that apply.
  □ Chemistry
  □ Physics
  □ Portuguese language
  □ English
  □ Mathematics
  □ Literature
  □ Philosophy
  □ Sociology
  □ History
  □ Geographic
  □ Biology
  □ Arts
7. What disciplines you liked least in the secondary education?*
  Check all that apply.
  □ Chemistry
  □ Physics
  □ Portuguese language
  □ English
  □ Mathematics
  □ Literature
  □ Philosophy
  □ Sociology
  □ History
  □ Geographic
  □ Biology
  □ Arts
8. Year you completed the secondary education (AAAA). *
  ________________________________
9. Did you attend preparatory course for entrance exam?
  Check only one.
  ◯ Yes
  ◯ No
10. In 2015/2016, what entrance exam you took in addition to the FICSAE exam?*
  Check only one.
  ◯ Yes
  ◯ No
11. How many entrance exams you did?*
  Check only one.
  ◯ One
  ◯ Two
  ◯ Three
  ◯ Four
  ◯ Five or more
12. Which university you enrolled for the entrance exam?*
  Check only one.
  ◯ FUVEST
  ◯ UNICAMP
  ◯ UNIFESP
  ◯ UNESP
  ◯ Other São Paulo States with its own entrance exam
  ◯ Other public universities/colleges out of the São Paulo State
  ◯ Other universities/colleges in São Paulo State that use the *Sisu* score - a unified selection system in Brazil
  ◯ Other private universities/colleges out of the São Paulo State
13. Did you move from your hometown to attend preparatory course for entrance exam?*
  Check only one.
  ◯ No
  ◯ Yes
14. Do you have any chronic disease?*
  Check only one.
  ◯ No
  ◯ Yes
15. How many hours, on average, you sleep daily?*
  Check only one.
  ◯ 1
  ◯ 2
  ◯ 3
  ◯ 4
  ◯ 5
  ◯ 6
  ◯ 7
  ◯ 8
  ◯ More than 8
16. Do you exercise?*
  Check only one.
  ◯ No
  ◯ Yes
17. What type of exercise do you do?*
  Check only one.
  ◯ Gym (weight training /collective exercise)
  ◯ Swimming
  ◯ Tennis
  ◯ Running/walking
  ◯ Basketball
  ◯ Handball
  ◯ Dancing
  ◯ Soccer
  ◯ Other
  I don't exercise
18. If you exercise regularly, what is the frequency (please, consider all exercises)*
  Check only one.
  ◯ Once a month
  ◯ Once every 15 days
  ◯ Once a week
  ◯ Two or three times a week
  ◯ Four to six times a week
  ◯ Everyday
  I don't exercise
19. What do you like to do in your leisure time?
  Check all that apply.
  □ Go to the movies
  □ Go to public parks
  □ Sports
  □ Dance club (bars/night clubs)
  □ Reading
  □ Watching TV
  □ Sleeping
  □ Visit relatives
  □ Go to live shows
  □ Go shopping
  □ Date with boyfriend/girlfriend
20. Are there physicians in your family?*
  Check only one.
  ◯ Yes
  ◯ No
21. If you have physicians in your family, what is your family's tie?
  Check all that apply.
  □ Father/mother
  □ Uncle/Aunt
  □ Grandparents
  □ Other
  □ There is no physician in my family.
22. What medical specialty do you intend to pursue?
  Check only one.
  I don't know
  ◯ Allergy and Immunology
  ◯ Cardiology
  ◯ Cardiovascular surgery
  ◯ Hand surgery
  ◯ Head and Neck Surgery
  ◯ Digestive surgery
  ◯ General surgery
  ◯ Pediatric surgery
  ◯ Plastic Surgery
  ◯ Chest surgery
  ◯ Vascular surgery
  ◯ General Medicine (Internal medicine)
  ◯ Dermatology
  ◯ Endocrinology and Metabology
  ◯ Endoscopy
  ◯ Gastroenterology
  ◯ Medical genetics
  ◯ Geriatrics
  ◯ Gynecology and Obstetrics
  ◯ Hematology and Hemotherapy
  ◯ Homeopathic
  ◯ Infectious diseases
  ◯ Mastology
  ◯ Family and community medicine
  ◯ Occupational medicine
  ◯ Sports medicine
  ◯ Physical and Rehabilitation medicine
  ◯ Intensive Care medicine
  ◯ Legal and Forensic medicine
  ◯ Nuclear medicine
  ◯ Preventive and social medicine
  ◯ Nephrology
  ◯ Neurosurgery
  ◯ Neurology
  ◯ Nutrology
  ◯ Ophthalmology
  ◯ Oncology
  ◯ Orthopedics and Traumatology
  ◯ Otorhinolaryngology
  ◯ Pathology
  ◯ Pediatrics
  ◯ Psychiatry
  ◯ Radiology and imaging diagnosis
  ◯ Radiotherapy
  ◯ Rheumatology
  ◯ Urology
  ◯ Other
  ◯ Outros
23. During your college education, do you intend to participate in other academic activities?
  Check only one.
  I don't intend
  ◯ Athletics
  ◯ Student academic center
  ◯ Junior scientific initiation
  ◯ Other:_________________________

FICSAE: *Faculdade Israelita de Ciências da Saúde Albert Einstein*; FUVEST: *Fundação Universitária para o Vestibular*; UNICAMP: *Universidade Estadual de Campinas*; UNIFESP: *Universidade Federal de São Paulo*; UNESP: *Universidade Estadual Paulista “Júlio de Mesquita Filho”.*
